# Triplet transfer from PbS quantum dots to tetracene ligands: is faster always better?†

**DOI:** 10.1039/d2tc03470k

**Published:** 2022-10-11

**Authors:** Victor Gray, William Drake, Jesse R. Allardice, Zhilong Zhang, James Xiao, Daniel G. Congrave, Jeroen Royakkers, Weixuan Zeng, Simon Dowland, Neil C. Greenham, Hugo Bronstein, John E. Anthony, Akshay Rao

**Affiliations:** Cavendish Laboratory, University of Cambridge J. J. Thomson Avenue Cambridge CB3 0HE UK victor.gray@kemi.uu.se ar525@cam.ac.uk; Department of Chemistry – Ångström Laboratory, Uppsala University Box 523 751 20 Uppsala Sweden; Department of Chemistry, University of Cambridge Lensfield Road Cambridge CB2 1EW UK; Cambridge Photon Technology J. J. Thomson Avenue Cambridge CB3 0HE UK; University of Kentucky Center for Applied Energy Research 2582 Research Park Dr Lexington Kentucky 40511 USA

## Abstract

Quantum dot-organic semiconductor hybrid materials are gaining increasing attention as spin mixers for applications ranging from solar harvesting to spin memories. Triplet energy transfer between the inorganic quantum dot (QD) and organic semiconductor is a key step to understand in order to develop these applications. Here we report on the triplet energy transfer from PbS QDs to four energetically and structurally similar tetracene ligands. Even with similar ligands we find that the triplet energy transfer dynamics can vary significantly. For TIPS-tetracene derivatives with carboxylic acid, acetic acid and methanethiol anchoring groups on the short pro-*cata* side we find that triplet transfer occurs through a stepwise process, mediated *via* a surface state, whereas for monosubstituted TIPS-tetracene derivative 5-(4-benzoic acid)-12-triisopropylsilylethynyl tetracene (BAT) triplet transfer occurs directly, albeit slower, *via* a Dexter exchange mechanism. Even though triplet transfer is slower with BAT the overall yield is greater, as determined from upconverted emission using rubrene emitters. This work highlights that the surface-mediated transfer mechanism is plagued with parasitic loss pathways and that materials with direct Dexter-like triplet transfer are preferred for high-efficiency applications.

## Abstract 2

We report the triplet energy transfer from PbS QDs to four energetically and structurally similar tetracene ligands, even with similar ligands we find that the triplet energy transfer dynamics can vary significantly.

## Graphical abstract

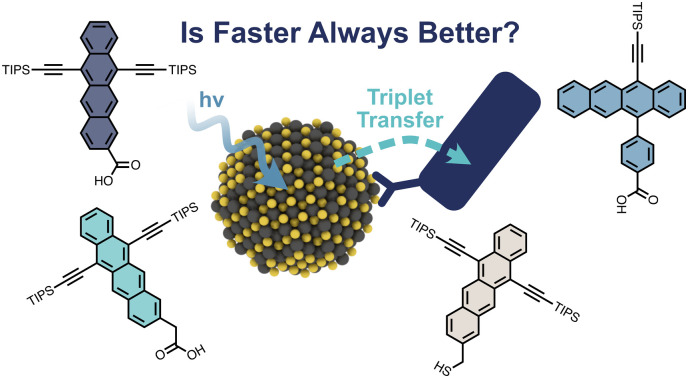

## Introduction

Organic–inorganic semiconductor nanocomposites often benefit from combining specific properties from each material type, resulting in materials with properties spanning a wider range of uses. For example, one class of organic–inorganic semiconductor nanocomposites exploit the different spin properties of organic and inorganic semiconductors. By combining a material with ill-defined spin states and a small exchange splitting (typically inorganic nanomaterials) with a material with well-defined spin states and a much larger exchange splitting (typically organic semiconductors), materials that are suitable for spin conversion and spin-mixing can be obtained. Recently these spin-mixing materials have gained increasing attention for applications such as solar energy harvesting, triplet sensitization, photon management and spin memories.^1–7^

**Fig. 1:**
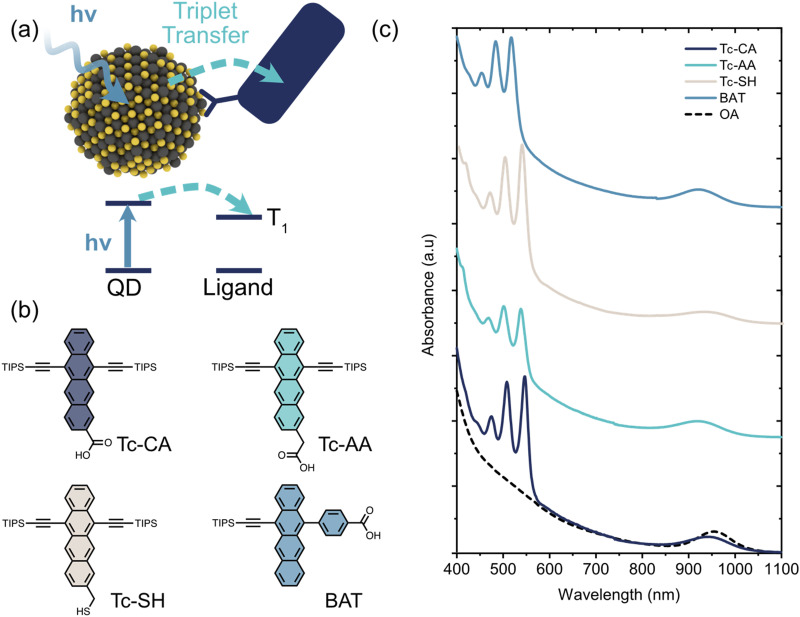
(a) Schematic illustration of the triplet sensitization process studied herein. A quantum dot (QD) absorbs a photon whereupon the exciton is transferred to an energetically available triplet state on a surface-anchored organic ligand. (b) Molecular structures of the four tetracene based ligands used as triplet acceptors. (c) Absorption spectra of the PbS/ligand QDs used in this study, compared to the as synthesized oleic acid (OA)-capped PbS QDs.

Different types of inorganic nanomaterials, such as perovskite nanocrystals and films,^8–15^ chalcogenide QDs and platelets,^1,16–25^ as well as CuInS_2_^26^ QDs have all been combined with organic semiconductors to achieve either singlet-to-triplet and triplet-to-singlet spin conversion. Due to the small exchange splitting in inorganic nanomaterials spin-mixing occurs in the inorganic part, with the organic semiconductor functioning as a triplet acceptor or donor depending on the direction of exciton transfer. Hence, a key part of the overall spin-mixing process is the transfer of a triplet exciton from (to) an inorganic nanomaterial to (from) the organic semiconductor. A schematic illustration of triplet exciton transfer from a photoexcited PbS QD to an organic ligand is shown in Fig. 1a.

Interestingly, the mechanism for triplet exciton transfer in these nanocomposites varies between structures, leading to some confusion. Recently, multiple studies have tried to address these issues by shedding light on what governs these different mechanisms of triplet exciton transfer. Currently, three different mechanisms are identified for triplet exciton transfer across organic–inorganic interfaces; The first is a direct Dexter type exchange mechanism where the whole exciton (electron and hole) transfers simultaneously across the organic–inorganic interface.^14,20^ The second is a stepwise charge-transfer-mediated mechanism that can occur if the band alignment is suitable.^14,15^ Here the hole (or electron) is first transferred across the interface, followed by the electron (hole) at a later time, completing the transfer. The third mechanism is also stepwise, however, in this case the intermediate state is found to be a surface trap state on the inorganic QD, localizing the hole to the surface before transfer to the organic semiconductor is completed.^27,28^

Usually, the studies detailing the different mechanisms cover one or two specific material combinations and comparison between studies is difficult as they often deal with structurally and energetically different materials. Hence, it can be difficult to draw general conclusions regarding what governs the mechanism. Therefore, we choose to study four structurally similar tetracene ligands attached to a high-energy PbS QD to better understand the differences in triplet transfer mechanisms from QD to ligand and how it relates to ligand structure. We chose to study ligands with different binding motifs (carboxylic acid and thiol), different spacers (no spacer, CH_2_ and phenyl) as well as different attachment points to the ligand core. We previously showed that SH anchors improve the thermal stability of the QD-ligand complex without affecting the triplet transfer efficiency of ligand to QD triplet transfer.^29^ Rigid spacers on the other hand have been show to decrease the triplet transfer efficiency.^30–32^ Here we observe a stepwise surface-trap-mediated triplet transfer mechanism in three of the QD/ligand systems and a direct, albeit slower, transfer in the fourth QD/ligand system. Interestingly, the slower direct mechanism results in a higher triplet transfer efficiency as manifested in a significantly higher triplet-sensitized upconversion (UC) emission from rubrene. Based on our observations we conclude that the surface-state-mediated transfer also leads to significant losses and should be avoided in high efficiency devices.

## Results and discussion

PbS quantum dots with an exciton absorption peak at 954 nm (1.3 eV) were synthesized as described previously (see ESI† for details). As triplet acceptor ligands we choose to investigate three 6,11-bis-triisopropylsilylethynyl tetracene (TIPS-Tc) derivatives and one monosubstituted 5-(4-benzoic acid)-12-triisopropylsilylethynyl tetracene (BAT) derivative, Fig. 1. The three TIPS-Tc derivatives have different anchoring groups; carboxylic acid (Tc-CA), acetic acid (Tc-AA) and thiol (Tc-SH), however all three derivatives attach to the QD from the tetracene short (pro-*cata*) side. The fourth derivative, 4-(12-((triisopropylsilyl)ethynyl)tetracen-5-yl)benzoic acid (BAT), on the other hand, has the benzoic acid anchoring group attached to the long (*peri*) side of the tetracene backbone. Ligand exchange was performed in a toluene/THF mixture following previously reported protocols and the ligand coverage was determined by UV/Vis absorption (Fig. 1 and Fig. S1, S2, ESI†). The ligand coverage varies from 10–40 ligands/quantum dot with the highest coverage for PbS/BAT, Table 1 and Fig. 1 and Fig. S1 (ESI†). Decreasing the ligand input during ligand exchange reduces the number of ligands attached to the QD, but the general trend between the four ligands is preserved (Fig. S1, ESI†). Four samples with the most comparable ligand coverage are chosen for further studies, however we also normalize determined rate constants to account for different ligand coverage.

**Table 1:** Properties of the PbS/Ligand systems studied herein

Ligand	*λ* #Ligand/QD^a^	*τ*_TET_ b (ns)	*k* ^0^_TET_ c (s^−1^)	*τ*_Ligend_ d(μs)	Relative UC Intensity^e^	*ϕ*_TET−A_ f (%)
Tc-CA	16.6	73.3 ± 3.6	9.7 ± 0.4 × 10^5^	60.1 ± 1.5	0.26	98.0
Tc-AA	10.2	114 ± 10	6.8 ± 0.5 × 10^5^	29.5 ± 1.6	0.05	95.7
Tc-SH	24.6	116 ± 9	3.3 ± 0.3 × 10^5^	2.9 ± 0.1	0.02	76.2
BAT	40.7	567 ± 102	3.7 ± 0.5 × 10^4^	148 ± 25	1	99.3

a Average number of ligands attached per QD, determined from UV-vis absorption measurements.

b Time constant for the rise of the triplet signal from single-exponential fits to ns-TA data.

c Intrinsic rate constant for triplet transfer to one ligand obtained assuming a Poisson distribution of bound ligands.

d Lifetime of the ligand triplet state, from fits to ns-TA data.

e Relative upconversion intensity for PbS/ligand sensitizers with 10 mM rubrene upon 785 nm excitation.

f Estimated triplet transfer efficiency for triplet transfer from ligands to rubrene in toluene, assuming *k*_TET_ = 1 × 10^8^ M^−1^ s^−1^.

To study the triplet transfer dynamics in the PbS/ligand systems we first turn to ultrafast transient absorption (TA). By exciting the PbS QD directly at 700 nm we follow the decay of the PbS ground state bleach (GSB). For the three ligands attached on the pro-*cata* side, Tc-CA, Tc-AA and Tc-SH, the PbS GSB decays significantly within the first 2 ns, Fig. S4 (ESI†). PbS/BAT, however, shows no decay on this timescale, similar to the as synthesized OA-capped QD. As the QD signal decays we see no growth of any other signals on this timescale and wavelength region. Our observations are in line with the stepwise triplet transfer dynamics observed for PbS/pentacene derivatives previously.^27,33^ In those studies, the pentacene triplet signal grew in at a later time scale compared to the decay of the QD GSB. We therefore continued with ns-resolved TA to investigate if the ligand triplet would be formed on the ns–μs timescale.

**Fig. 2:**
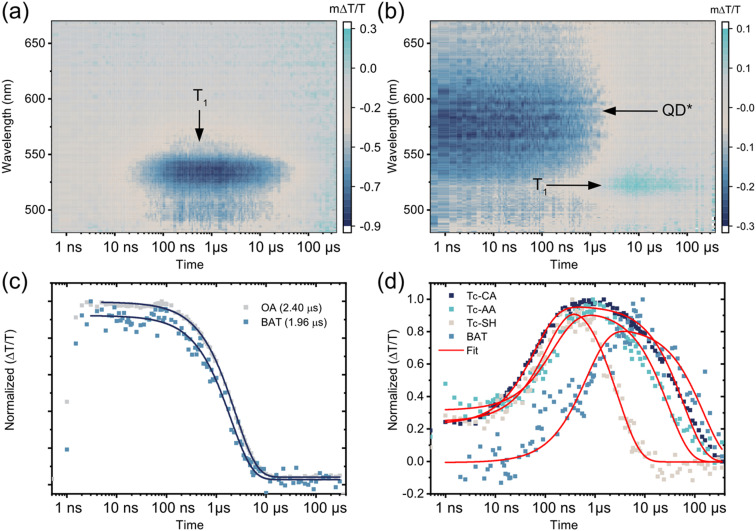
(a) ns-resolved transient absorption (TA) 2D map of PbS/Tc-CA showing no QD features but a delayed formation of Tc-CA triplet photoinduced absorption (PIA) at 525–550 nm. (b) ns-resolved TA 2D map of PbS/BAT with QD PIA features at 525–625 nm decaying and a correlated formation of BAT ground state bleach (GSB) at 525 nm. (c) Comparison of the normalized transient absorption signal of PbS/OA at the GSB (950 nm) with the GSB signal of PbS/BAT showing a 20% quenching of the QD lifetime. (d) Comparison of the ligand triplet formation dynamics. For Tc-CA, Tc-AA and Tc-SH the dynamics is monitored directly at 525–535 nm. For BAT the dynamics at 520–530 nm is corrected by removing contribution of the QD PIA decay. Fits are a rise term consisting of a sum of exponential decays assuming a Poisson distribution of bound ligands and a monoexponential decay component. All samples were excited at 700 nm with 400 nJ per pulse.

Fig. 2 compares the ns-TA 2D maps of PbS/Tc-CA and PbS/BAT with evidence of triplet formation on these timescales. For PbS/Tc-CA and the other two ligands attached on the pro-*cata* side, Tc-AA and Tc-SH, no QD features are observed in the visible or NIR region, in line with the fast decay on the fs–ps timescale, Fig. 2(a) and Fig. S4 (ESI†). After 10 ns the growth of a photoinduced absorption (PIA) around 530 nm is observed, peaking at a few 100 ns. The PIA feature has been reported by us and others previously and is evidence of the Tc-CA ligand triplet state.^25,34^ Similar dynamics are observed for PbS/Tc-AA and PbS/Tc-SH, Fig. S5 (ESI†).

As was the case with the ps TA dynamics, the dynamics of PbS/BAT on the ns–μs timescale differs significantly from the other three PbS/ligand systems. Firstly, the QD PIA in the visible region and the GSB in the NIR region are both clearly observable at the initial time. Compared to PbS/OA the QD lifetime is reduced by about 20%, from 2.40 μs to 1.96 μs, with BAT attached, Fig. 2c. Secondly, as the QD signals decay there is a concomitant growth of the BAT GSB around 525 nm, indicating direct triplet transfer to BAT.

To quantify the triplet transfer dynamics in more detail the kinetics of the PbS/ligand systems were inspected. The kinetics of PbS/Tc-CA, PbS/Tc-AA and PbS/Tc-SH were extracted by averaging the signal in the 525–535 nm region, Fig. 2d. For PbS/BAT the signals from the ligand and QD are overlapping in the 520–530 nm region. However, as the GSB signal of the QD in the NIR region is free of interfering signals from the ligand, the pure QD dynamics can be extracted. The obtained QD dynamics are then subtracted from the superimposed kinetics in the 520–530 nm region, yielding the ligand-only kinetics shown in Fig. 2d.

Following the procedure by Morris-Cohen *et al.*^35^ we extract the intrinsic triplet transfer per ligand (*k*^0^_TET_) assuming a Poisson distribution of bound ligands with the average number of ligands per QD (*λ*) (See ESI† for details). The triplet life time (*τ*_Ligand_) of the bound ligand was also extracted from the fitting procedure assuming a monoexponential decay. The extracted triplet transfer rates, *k*^0^_TET_ and life times are summarised in Table 1. The triplet transfer to Tc-CA, Tc-AA and Tc-SH occurs in 70–120 ns and the intrinsic triplet transfer rate per ligand (*k*^0^_TET_) are 9.7 × 10^5^, 6.8 × 10^5^ and 3.3 × 10^5^ s^−1^, respectively. It can be noted that with the increase in distance between QD and ligand by one saturated carbon for Tc-CA and Tc-AA the intrinsic triplet transfer rate slightly decreases. Tc-SH which should have a similar distance to Tc-AA is slower still. A decrease in triplet transfer with increasing separation is expected,^30,31^ although not always observed for short distances.^36^ Even though the rate of transfer for Tc-CA, Tc-AA and Tc-SH differs slightly, the mechanism for triplet transfer occurs in a similar manner. However, as highlighted above, the triplet transfer to BAT is fundamentally different. The triplet transfer to BAT occurs in 567 ns and the intrinsic triplet transfer rate *k*^0^_TET_ is 3.7 × 10^4^ s^−1^. Even though it is expected that *k*^0^_TET_ for BAT is slower, due to the increase in distance associated with the benzoic acid binding group, the fact that PbS/BAT shows minor QD quenching compared to the other three ligands indicates that two different mechanisms are at play.

Both direct^14,20,21^ and stepwise^14,15,27,28,33^ triplet transfer from QDs to organic ligands have been observed previously. What governs the balance between the different mechanisms is still an area of debate and ongoing research. However, the most recent observations point at two different stepwise mechanisms and one direct Dexter-like mechanism. Depending on the binding geometry of the ligand and the extent of quantum confinement of the QD, the direct Dexter-like mechanism has been shown to be mediated by different coupling mechanisms:^37^ For highly quantum confined QDs and *peri*-substituted acene ligands Dexter transfer is mediated by through space coupling whereas pro-*cata* substituted acenes are better suited for less quantum confined QDs where transfer is mediated by a through bond coupling.^37^ The direct mechanism has further been suggested to possibly be mediated by a virtual charge-transfer state.^14^ On the other hand, for the two stepwise mechanisms the difference is that they invoke two different intermediate states. The first mechanism, referred to as the charge-transfer-mediated triplet transfer, has been shown to occur when the ligand and QD band alignment favours hole or electron transfer.^14,15,33^ In this case, the hole (electron) is transferred first, forming a charge-separated intermediate state, before the electron is transferred to complete the triplet exciton transfer. In the second mechanism the QD exciton is rapidly tapped at a surface state, followed by triplet transfer to the ligand.^27,28^

**Fig. 3:**
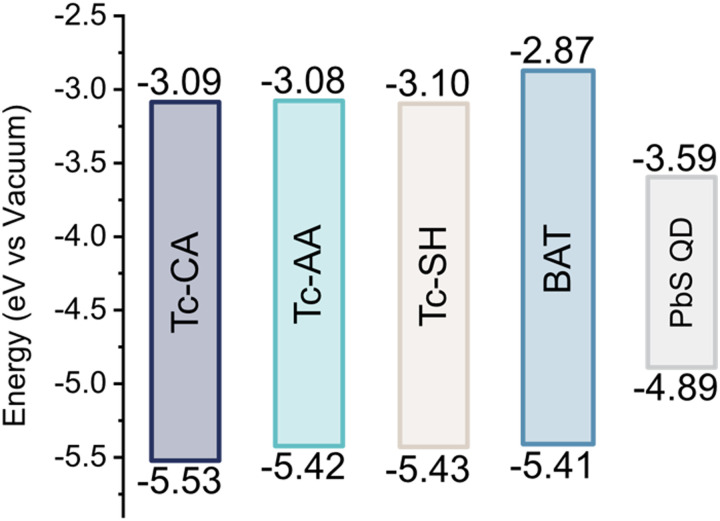
HOMO and LUMO levels of the ligands and PbS/OA QDs. Determined from cyclic voltammetry relative to ferrocene.

To differentiate between the possible mechanisms, it is important to have a good understanding of the HOMO and LUMO levels of the ligand relative to the valence and conduction bands of the QD. We therefore turned to cyclic voltammetry to determine these, Fig. S6–S8 (ESI†). Interestingly the HOMO of the four ligands is very similar, Fig. 3, and the four ligands have equally poor alignment for hole-mediated triplet transfer. We also performed DFT calculations of the neutral and deprotonated ligands to corroborate our experimental findings, and estimate the triplet energy of all ligands, see the ESI† Fig. S9–S12 and Table S1 for details. The calculated frontier orbital energies for both the neutral and deprotonated ligands match well with the measured HOMO and LUMO levels. Only Tc-SH show any significant difference between the neutral and deprotonated form, where the HOMO–LUMO gap decreases as the HOMO rises in energy enough to open up for hole transfer from the QD, Fig. S9 (ESI†). However, as the Tc-SH ligand absorption spectrum when bound to the PbS QD is unchanged compared to the free form, we conclude that the neutral form is a better description of the bound ligand. Hence, both experimental and calculations indicate that hole transfer between PbS QDs and the bound ligands is improbable. It should be noted that the surface ligands can alter the valence and conduction bands of the QDs, which might open up for possible hole transfer.^38^ However, since the ligand loading of the active ligand is relatively small compared to the native oleic acid coverage, and that the ligands themselves are energetically similar, we assume that the influence of the tetracene ligands on the PbS conduction and valence bands is small enough for us to disregard this effect. Ruling out hole-transfer-mediated triplet transfer would suggest that the stepwise mechanism is governed by surface states on the QD. Triplet transfer to the BAT ligand on the other hand occurs *via* direct triplet transfer as concluded above. The estimated triplet energies are reported in Table S1 (ESI†). The calculated T_1_ for Tc-CA is about 0.1 eV lower than that previously determined experimentally.^25^ The triplet energies are similar between all ligands, with BAT having a 0.1 eV higher triplet energy, in accordance with the higher S_1_ energy. Hence, the difference in energy of the ligand triplets is unlikely to explain the difference in mechanism between the ligands.

The Tang group has readily studied *peri*-substituted tetracene ligands similar to BAT (*e.g.* tetracene-5-carboxylic acid, 5CT).^19,32,39,40^ Interestingly, triplet transfer to 5CT and its derivatives have all been through direct Dexter transfer,^32,39,40^ whereas pro-*cata* substituted pentacenes have displayed stepwise triplet transfer.^27,33^ Tang and co-workers have determined the normalized rate of triplet transfer to 5CT from PbS as ∼2 × 10^8^ s^−1^,^40^ 3–4 orders of magnitude greater than that observed for the ligands here. These differences can be rationalized in the following ways. First, since PbS QDs are highly quantum confined, *peri*-substituted ligands like 5CT are expected to result in the strongest coupling.^37^ Secondly, compared to the structurally similar BAT, 5CT is significantly closer to the QD surface as it lacks the phenyl-spacer. For example, 5CPT that also has an phenyl spacer has a normalized triplet transfer rate of 9 × 10^5^ s^−1^,^32^ still greater than that for BAT, but significantly slower than 5CT. Thirdly, a trap state mediated triplet transfer as observed for Tc-CA, Tc-AA and Tc-SH, is expected to be significantly slower compared to direct transfer,^41^ as observed for 5CT.

What governs the change in mechanism is still unclear. Based on our findings and the results discussed above for other acene ligands it seems that the binding geometry is the determining factor. Wu *et al.* observed that the binding geometry significantly influenced the coupling between QD and ligand,^37^ however this is not sufficient to explain the change in mechanism observed here. Our current hypothesis is that the surface states are introduced during ligand exchange, and that the different binding geometries and local size of the ligand expels the native oleic acid ligands differently, resulting in trap states for the pro-*cata* substituted ligands, but not for *peri*-substituted ligands such as BAT and 5CT. These directions will be explored in future research.

## Triplet–Triplet annihilation upconversion as a measure of triplet transfer efficiency

We continue our study by investigating the efficiency of triplet energy transfer in all four systems. Only 20% of the QD exciton population was quenched by BAT, whereas 100% of the QD population was quenched when the other three ligands were attached. That could suggest that Tc-CA, Tc-AA and Tc-SH are more efficient as triplet acceptors. We therefore used these PbS/ligand systems as sensitizers for NIR-to-visible photon upconversion based on triplet–triplet annihilation^1,6,42–44^ to get an indirect measure of the triplet yield.

In triplet–triplet annihilation upconversion a triplet sensitizer absorbs low-energy photons and subsequently transfers the triplet energy to an annihilator molecule. The triplet excited annihilator then interacts with another triplet excited annihilator to fuse the energy, forming a singlet excited annihilator and a ground state annihilator. The singlet excited annihilator can then emit a high-energy photon when returning to its ground state. The upconversion quantum yield (*ϕ*_UC_), *i.e.* the number of photons emitted by the annihilator per absorbed photons by the sensitizer can be described by eqn (1):

1 *ϕ*_UC_ = *ϕ*_TET−L_*ϕ*_TET−A_*ϕ*_TTA_*ϕ*_F_

where *ϕ*_TET−L_ is the triplet transfer efficiency from QD to ligand, *ϕ*_TET−A_ is the triplet transfer efficiency from ligand to annihilator, *ϕ*_TTA_ is the triplet–triplet annihilation quantum yield and *ϕ*_F_ is the fluorescence quantum yield of the annihilator.

**Fig. 4:**
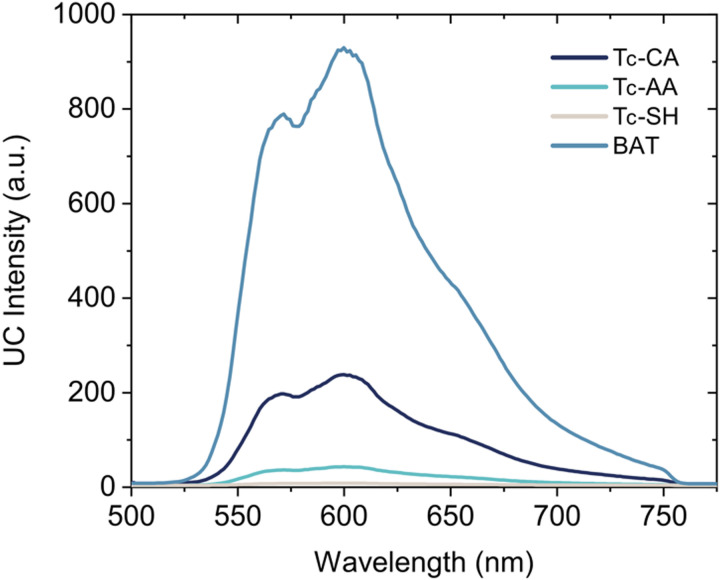
Upconverted anti-Stokes emission upon 785 nm excitation of samples containing PbS/Ligand and 10 mM Rubrene in oxygen free toluene. Laser scatter is filtered by a 750 nm short pass filter.

By mixing the PbS/ligand sensitizers with rubrene, a common annihilator molecule, in deaerated toluene, upconverted yellow emission is observed upon excitation with a 785 nm CW laser for all PbS/ligand sensitizers. Interestingly, PbS/BAT outperformed the other three PbS/ligand sensitizers displaying 5 times greater upconversion emission intensity than the second best PbS/Tc-CA sensitizer, Fig. 4.

From eqn (1) it is understood that the observed upconversion intensity depends on the efficiency of a number of processes. As we use the same annihilator and concentration in the samples *ϕ*_F_ is the same across all samples. Furthermore, *ϕ*_TTA_ will depend on the concentration of rubrene triplets, which relates to the excitation power and the efficiency of the two triplet transfer processes.^45,46^ However, for a given triplet concentration, *ϕ*_TTA_ is an intrinsic property of the annihilator, hence a reduction in *ϕ*_UC_ due to a reduction in *ϕ*_TTA_ across the samples would be directly linked to the two triplet transfer processes. Hence, it remains to elucidate if the reduction in the overall triplet transfer originates from the triplet transfer from QD to ligand (TET-L), or from ligand to the annihilator rubrene (TET-A). The triplet transfer from ligand to rubrene is a bimolecular process which depends on both the rubrene concentration and triplet lifetime of the ligand. Assuming a bimolecular triplet transfer rate to rubrene of *ϕ*_TET−A_ = 1 × 10^8^ M^−1^ s^−1^, a reasonable rate considering triplet transfer to rubrene in similar systems,^47,48^ we can estimate the triplet transfer efficiency *ϕ*_TET−A_ from the ligand lifetime and rubrene concentration [Rub] according to eqn (2):2
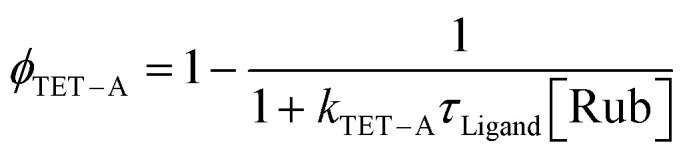


With the rubrene concentration at 10 mM the bimolecular triplet transfer efficiency to rubrene is estimated to be >95% for Tc-CA, Tc-AA and BAT, and ∼75% for Tc-SH due to the shorter triplet lifetime (Table 1). In fact, for the more than 5 times lower upconversion intensity of the pro-*cata* anchored ligands to be explained by the reduction in diffusional triplet transfer the bimolecular rate constant, *k*_TET−A′_ for the pro-*cata* ligands must be as low as 10^5^ M^−1^ s^−1^, see the ESI† for further details. For example, even for *k*_TET−A_ = 0.4 × 10^8^ M^−1^ s^−1^ the triplet transfer efficiency to rubrene is >90% for all but Tc-SH. Based on these estimates, we conclude that the main reason for the difference in observed upconversion emission intensity stems directly from the difference in triplet transfer from QD to ligand, *ϕ*_TET−L_. Hence, the upconversion intensity is therefore a relative measure of the triplet population of the ligands. The difference in upconversion intensity in these samples highlights an interesting finding in these PbS/ligand systems: more triplets are transferred to BAT than the other three ligands, even though the transfer rate is slower. We can therefore conclude that the direct mechanism which governs triplet transfer in PbS/BAT does not introduce significant loss pathways, whereas a surface mediated mechanism, as in the cases for PbS/Tc-CA, PbS/Tc-AA and PbS/Tc-SH is plagued by parallel loss pathways competing with the triplet transfer.

## Conclusions

In summary, we have studied the triplet transfer from PbS QDs to organic semiconductors in four QD/ligand systems where the ligand is a TIPS-tetracene derivative with either a carboxylic acid (Tc-CA), acetic acid (Tc-AA), methanethiol (Tc-SH) or benzoic acid (BAT) anchor group. We find that for the three first ligands, substituted on the short pro-*cata* side, triplet energy occurs through a stepwise mechanism. Based on the band alignment we rule out hole-mediated triplet transfer,^14,15,33^ which suggests a QD surface-state-mediated triplet transfer mechanism.^27,28^ In the PbS/BAT system, where the ligand is anchored *via* the long *peri* side, direct Dexter-like triplet energy transfer is observed. Considering the energetic similarities between all four ligands the major difference between the Tc-CA/Tc-AA/Tc-SH and BAT ligand is the binding geometry. How the binding geometry might influence the triplet transfer mechanism will be investigated in future work, but it might relate to previous observations of *peri* substitutions in pentacene having a greater impact on the electronic nature of the acene core compared to pro-*cata* substitution.^49^ However, from triplet–triplet annihilation photon upconversion measurements we can conclude that the direct triplet transfer mechanism, even though slower, is more efficient, suggesting that the surface-mediated transfer is plagued with parasitic loss pathways. Therefore, materials with direct Dexter-like triplet transfer are preferred for high-efficiency applications.

## Conflicts of interest

There are no conflicts to declare.

## Supplementary Material

TC-010-D2TC03470K-s001
