# Supplementary material for: Triplet transfer from PbS quantum dots to tetracene ligands: is faster always better?
Source: J Mater Chem C Mater. 2022 Oct 11;10(43):16321–9. doi: 10.1039/d2tc03470k (PMC9648495; doi:10.1039/d2tc03470k)
Supplement: TC-010-D2TC03470K-s001 [file TC-010-D2TC03470K-s001.pdf]

Electronic Supplementary Material (ESI) for Journal of Materials Chemistry C.
This journal is © The Royal Society of Chemistry 2022

Supporting Information for: Triplet Transfer from PbS Quantum dots to Tetracene Ligands: Is Faster Always Better?

Victor Gray[†,‡*], William Drake[†], Jesse R. Allardice[†], Zhilong Zhang[†], James Xiao[†], Daniel G. Congrave[¶], Jeroen Royakkers[¶], Weixuan Zeng[¶], Simon Dowland[#], Neil C. Greenham[†], Hugo Bronstein[¶], John E. Anthony[‖], Akshay Rao[†*]

[†]Cavendish Laboratory, University of Cambridge, J. J. Thomson Avenue, Cambridge CB3 0HE, U.K.

[‡]Department of Chemistry - Ångström Laboratory, Uppsala University, Box 523, 751 20, Uppsala, Sweden.

[¶] Department of Chemistry, University of Cambridge, Lensfield Road, Cambridge, CB2 1EW, U.K.

[#] Cambridge Photon Technology, J. J. Thomson Avenue, Cambridge, CB3 0HE, UK

[‖] Department of Chemistry, University of Kentucky, 161 Jacobs Science Building, Lexington Kentucky 40506-0174, United States.

*Corresponding author

Contact email: victor.gray@kemi.uu.se, ar525@cam.ac.uk

Table of Contents

***Experimental Methods*......2**

Chemicals......2

Synthesis......3

Dimethyl 2-(6,11-bis((triisopropylsilyl)ethynyl)tetracen-2-yl)malonate......3

2-(6,11-Bis((triisopropylsilyl)ethynyl)tetracen-2-yl)acetic acid (**Tc-AA**)......5

5-(4-benzenecarboxaldehyde)-12-triisopropylsilylethynyl tetracene......6

4-(12-((triisopropylsilyl)ethynyl)tetracen-5-yl)benzoic acid (**BAT**)......7

Quantum Dot Synthesis 8

Ligand Exchange 8

ps-Transient Absrobtion 10

ns-Transient Absorption 10

Cyclic Voltammetry 11

Additional ps-Transient Absorption Data 11

Additional ns-Transient Absorption Data 12

Cyclic Voltammograms 12

Calculations 13

Structure optimization and excited state analysis 13

Diffusional Triplet Energy Transfer 16

References 17

Experimental Methods

Chemicals

(8-bromotetracene-5,12-diyl)bis(ethyne-2,1-diyl)bis(triisopropylsilane) (Tc-Br), 6,11-bis(triisopropylsilyl)ethynyl)tetracene-2-carboxylic acid (Tc-CA) and (6,11-bis((triisopropylsilyl)ethynyl)tetracen-2-yl)methanethiol (Tc-SH) were synthesized as described previously.[1–3] All solvents were purchased in bulk from VWR. Anhydrous THF was purchased from Sigma Aldrich. All other chemicals were purchased from commercial sources and used as received unless otherwise noted. Silica was purchased from Silicycle. NMR spectra were measured on a 400 MHz Bruker Avance NEO spectrometer with Smart Probe. Chemical

shifts of each spectrum are reported in ppm and referenced to their corresponding deuterated solvents as listed. GC-MS was measured using a Bruker Scion-SQ GC-MS with an EI source. LD-MS measurements were acquired on a Bruker Microflex instrument. X-ray diffraction data were collected at low temperature on a Bruker D8 Venture kappa-axis diffractometer using MoK(alpha) X-rays

Synthesis

Dimethyl 2-(6,11-bis((triisopropylsilyl)ethynyl)tetracen-2-yl)malonate

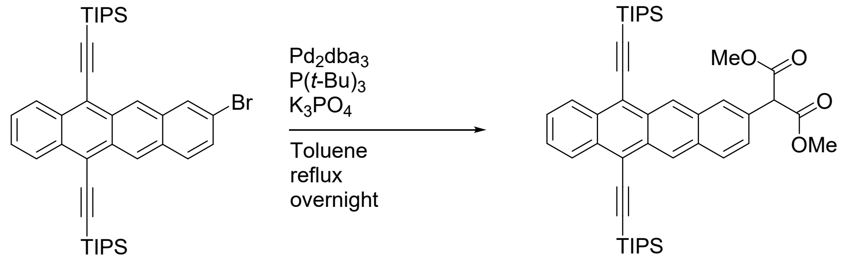

((8-bromotetracene-5,12-diyl)bis(ethyne-2,1-diyl))bis(triisopropylsilane) (**Tc-Br**) (334 mg, 0.50 mmol, 1.00 eq.), K_3PO_4 (318 mg, 1.50 mmol, 3.00 eq.) and Pd_2dba_3 (24 mg, 25 μmol, 5 mol%) were combined in an oven dried flask that was evacuated and backfilled with argon three times. Dry, degassed toluene (15 mL) was added, followed by dimethyl malonate (80 mg, 0.6 mmol, 1.20 eq.) and the resulting mixture was degassed for 15 min. P(*t*-Bu)$_3$ (1 M in toluene, 0.1 mL, 20 mol%) was next added and the mixture heated to reflux overnight. The solvent was removed under reduced pressure and the residue purified by flash chromatography on silica gel (eluent: 0→50% volume DCM in *n*-hexane) and the last pink/red band to elute was collected. The residue was suspended in methanol (20 mL), sonicated, and cooled in a freezer (*ca.* −20 °C) overnight. Filtration afforded dimethyl 2-(6,11-bis((triisopropylsilyl)ethynyl)tetracen-2-yl)malonate as a red powder (214 mg, 0.30 mmol, 60%). ^{1}H NMR (400 MHz, $CDCl_3$) δ (ppm) = 9.32 (s, 1H), 9.30 (s, 1H), 8.68 – 8.62 (m, 2H), 8.05 (d, J = 9.0 Hz, 1H), 7.94 (s, 1H), 7.58 (dt, J = 6.6, 3.0 Hz, 3H), 4.92 (s, 1H), 3.84 (s, 5H), 1.38 – 1.32 (m, 38H); ^{13}C NMR (101 MHz, $CDCl_3$) δ (ppm) = 168.4, 132.8, 132.7, 131.6, 131.4, 130.6, 130.5, 130.3, 129.2, 129.2, 127.4

(2C), 126.8 (2C), 126.6, 126.5, 126.3, 118.7, 118.7, 106.1, 106.0, 103.8, 103.8, 58.1, 53.0, 19.0, 18.9, 11.6, 11.6; HRMS (ASAP): *m/z* 719.3945 [M–H$^+$]. Calcd. for $C_{45}H_{59}O_4Si_2^+$: 719.3952.

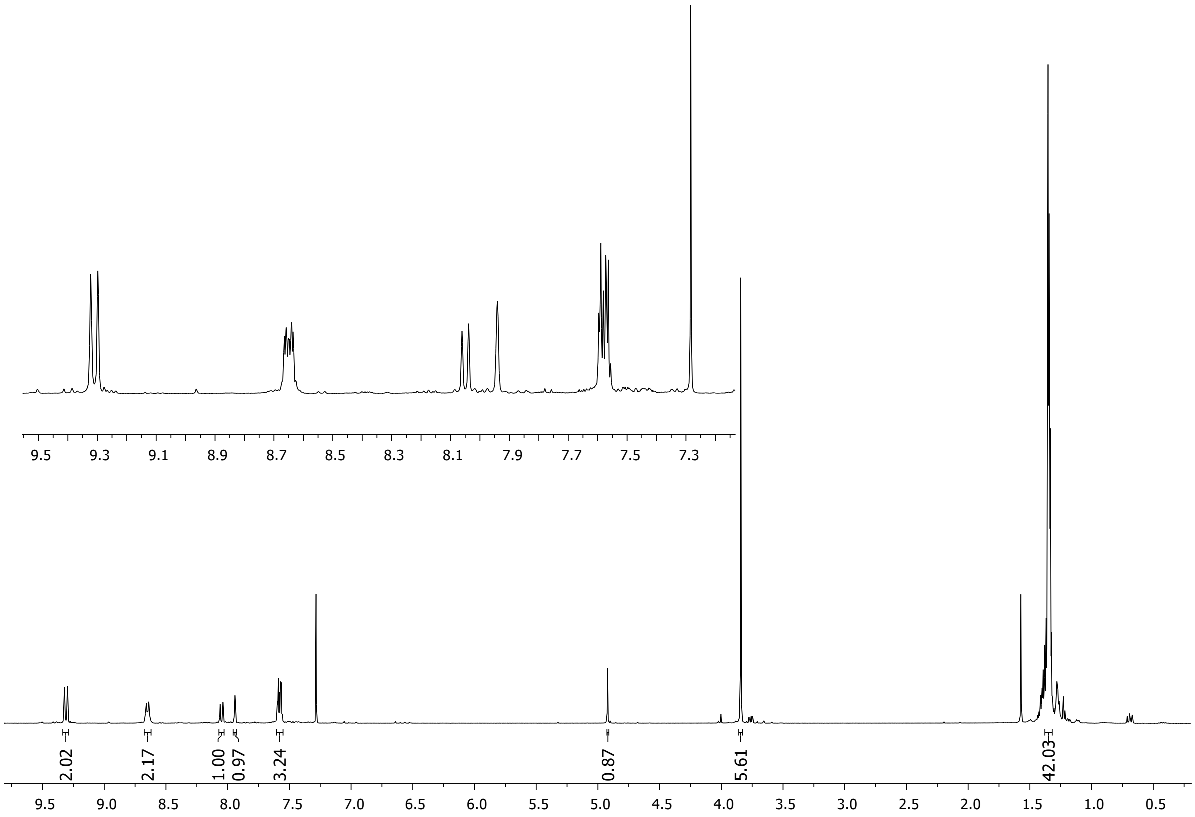

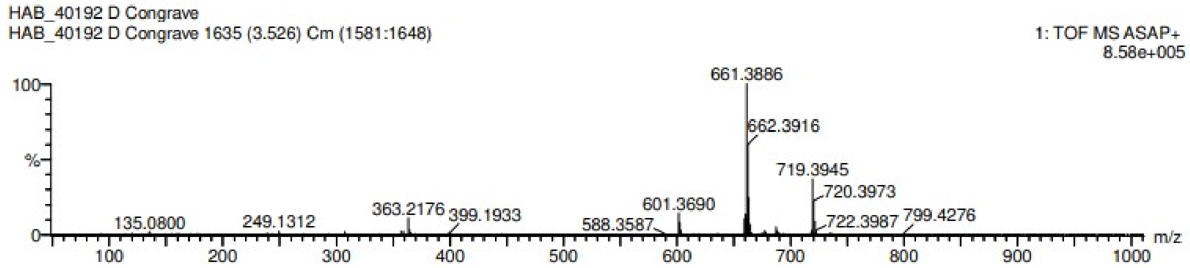

Minimum:				-1.5				
Maximum:		5.0	5.0	50.0				
Mass	Calc. Mass	mDa	PPM	DBE	i-FIT	Norm	Conf(%)	Formula
719.3945	719.3952	-0.7	-1.0	18.5	2157.2	n/a	n/a	C45 H59 O4 Si2

*2-(6,11-Bis((triisopropylsilyl)ethynyl)tetracen-2-yl)acetic acid (***Tc-AA)**

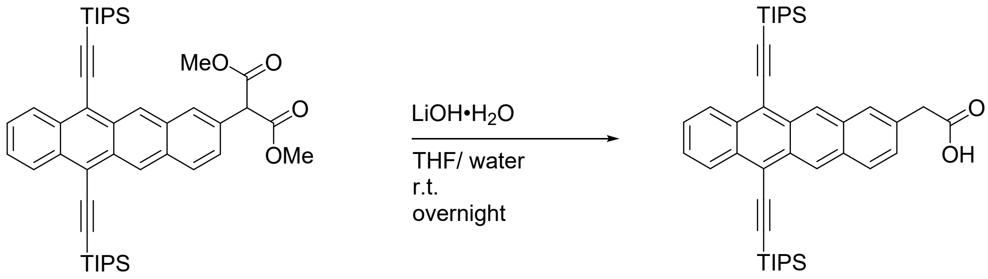

Dimethyl 2-(6,11-bis((triisopropylsilyl)ethynyl)tetracen-2-yl)malonate (173 mg, 0.24 mmol, 1.00 eq.) and lithium hydroxide monohydrate (50 mg, 1.20 mmol, 5.00 eq.) were dissolved in a mixture of THF (10 mL) and water (10 mL) and stirred at room temperature overnight. The mixture was then poured into 1 M HCl (100 mL) and extracted with DCM (3 × 20 mL). The extracts were combined, dried over $MgSO_4$, filtered and the solvent removed under reduced pressure. The residue was purified by flash chromatography on silica gel (eluent: gradient 0→20% volume EtOAc in DCM). The residue was suspended in methanol (10 mL), sonicated, and cooled in a freezer (ca. −20 °C) overnight. Filtration afforded dimethyl **Tc-AA** as a red powder (75 mg, 0.12 mmol, 50%). ^{1}H NMR (400 MHz, Acetone) δ (ppm) 10.88 (bs, 1H), 9.38 (s, 1H), 9.35 (s, 1H) 8.74 – 8.67 (m, 2H), 8.09 (d, J = 8.8 Hz, 1H), 8.04 (s, 1H), 7.70 (dd, J = 6.8, 3.2 Hz, 2H), 7.59 (d, J = 8.8 Hz, 1H), 3.93 (s, 2H), 1.42 – 1.32 (m, 42H); ^{13}C NMR (100 MHz, CD_2Cl_2) δ 174.8, 132.6, 132.5, 131.9, 131.5, 131.2, 130.4, 130.3, 128.8, 128.2, 128.0, 127.3 (2C), 126.9, 126.8, 126.2, 125.9, 118.6, 118.5, 106.3, 106.3, 103.6, 103.6, 40.8, 18.7, 11.6; HRMS (ASAP): m/z 647.3719 [M–H^+]. Calcd. for $C_{42}H_{55}O_2Si_2^+$: 647.3741.

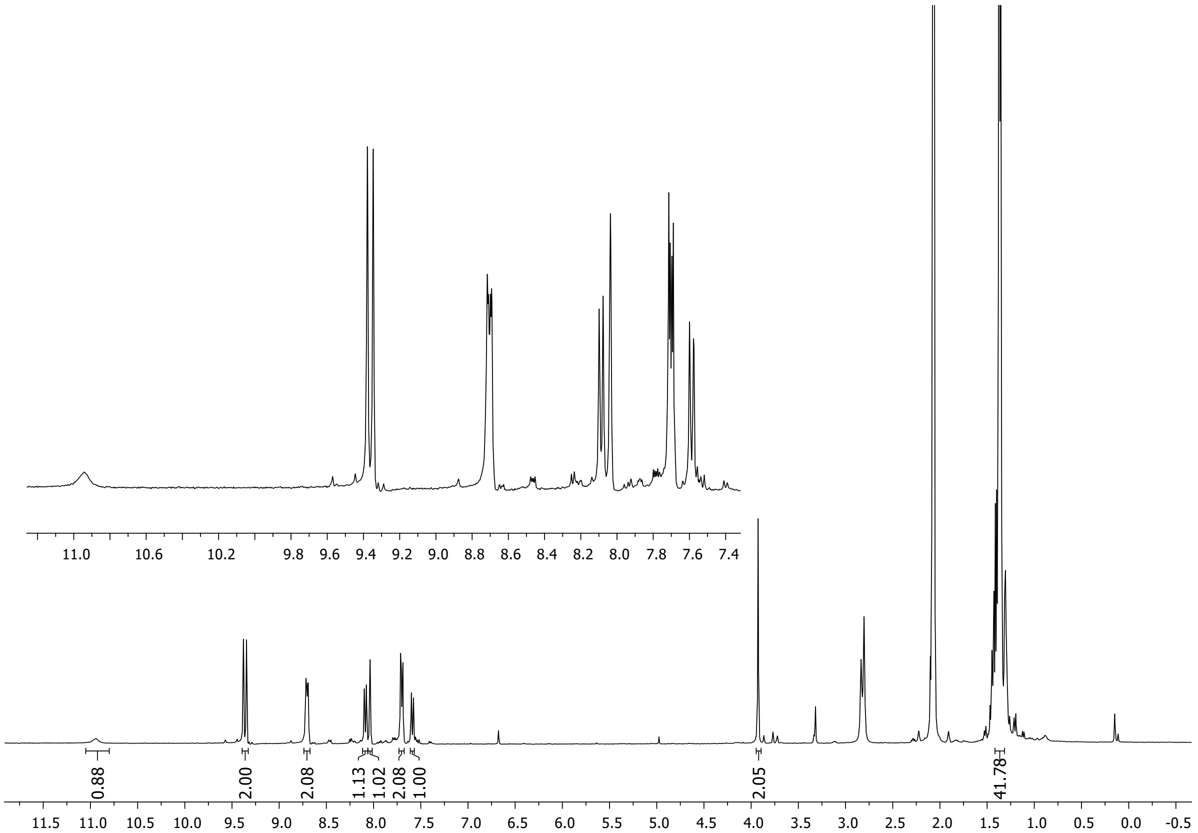

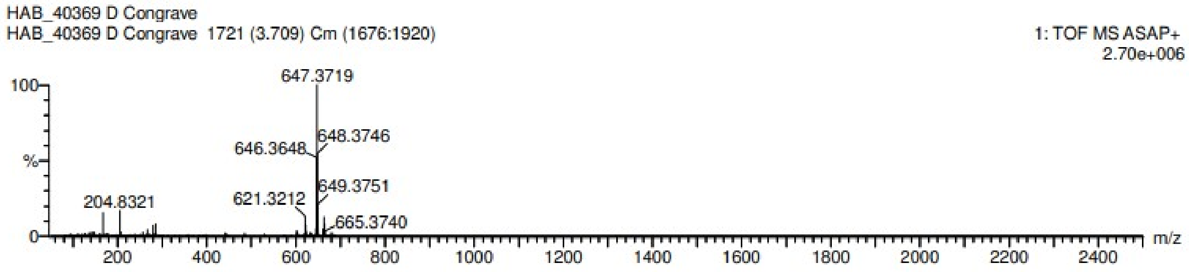

Mass	Calc. Mass	mDa	PPM	DBE	i-FIT	Norm	Conf(%)	Formula
Minimum:				-1.5				
Maximum:		5.0	5.0	50.0				
647.3719	647.3741	-2.2	-3.4	17.5	3439.8	n/a	n/a	C42 H55 O2 Si2

5-(4-benzenecarboxaldehyde)-12-triisopropylsilylethynyl tetracene

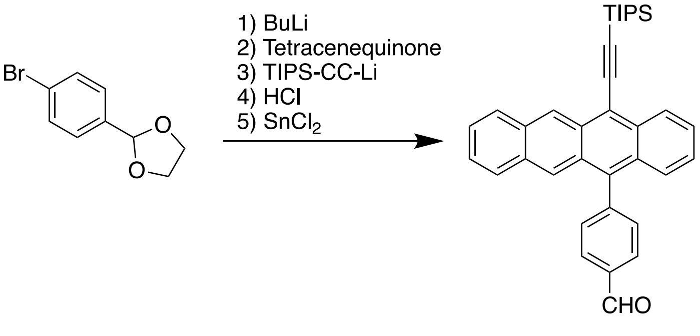

To a solution of 4-bromobenzaldehyde ethylene acetal (2.29g, 10 mmol) in 60 mL anhydrous THF cooled to -78 °C was slowly added 4 mL (10 mmol of a 2.5M solution in hexanes) of n-BuLi. After stirring for 20 minutes at -78 C, 2.5 g (9.8 mmol) of naphthacenequinone was added to the solution, which was then allowed to warm slowly to room temperature. After 3 hours, 12 mmol of prepared solution of litiho triisopropylsilyl acetylene was added to this solution, and the reaction mixture was stirred at room temperature overnight. 10 mL of 10% aqueous HCl was then added, and the solution stirred a further 24 hours. Finally, 2 grams of stannous chloride dihydrate was added to the mixture, which was stirred for a further two hours. The solution was poured into hexanes, extracted three times with water, dried with anhydrous magnesium sulfate, and passed through a thin pad of silica gel (flushed with hexanes, then 1:1 hexanes:dichloromethane) to remove baseline impurities. The compound was purified by chromatography on silica gel (hexanes -> 5:1 hexanes:dichloromethane) and recrystallization from hexanes to yield 2.3 g (45%) of aldehyde product. ^{1}H NMR ($CDCl_3$, 400 MHz) ∂ 10.229 (s, 1H), 9.399 (s, 1H), 8.707 (d, J = 5 Hz, 1H), 8.158 (d, J = 6 Hz, 2H), 8.012 (d, J = 5 Hz, 1H), 7.777 (d, J = 5 Hz, 1H), 7.654 (d, J = 6 Hz, 2H), 7.391, (br m, 6H), 1.346 (br s, 21H). MS (LD): 512 (M^+). HRMS calculated for $C_{36}H_{36}OSi$: 512.2535; found: 512.2533. The structure was also confirmed by X-ray crystallography.

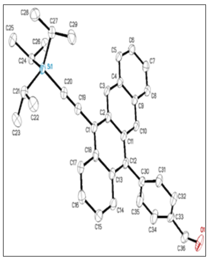

*4-(12-((triisopropylsilyl)ethynyl)tetracen-5-yl)benzoic acid (***BAT***)*

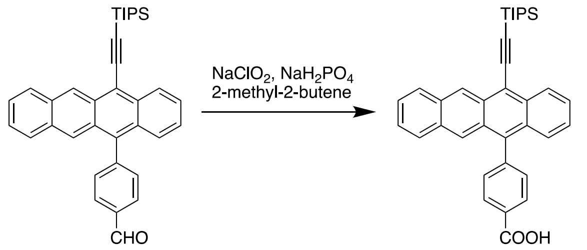

To a solution of 1 gram (2 mmol) of aldehyde in 50 mL THF and 5 mL 2-methyl-2-butene was added a solution of 1.19 g (13 mmol) $NaClO_2$ and 1.58 g (13 mmol) NaH_2PO_4 dissolved in 10 mL water. The reaction was monitored by TLC until all starting material had been consumed (~4 h). The solution was poured into dichloromethane and extracted 3 times with water. The organic layer was dried over magnesium sulfate, filtered, and evaporated. The product was purified chromatographically (silica gel, dichloromethane -> 9:1 dichloromethane:THF) to yield 0.89 grams (85%) of the desired carboxylic acid as a red solid. . ^1H NMR ($CDCl_3$, 400 MHz) ∂ 9.401 (s, 1H), 8.711 (d, J = 5 Hz, 1H), 8.153 (d, J = 6 Hz, 2H), 8.009 (d, J = 5 Hz, 1H), 7.762 (d, J = 5 Hz, 1H), 7.649 (d, J = 6 Hz, 2H), 7.389, (br m, 6H), 1.355 (br s, 21H). MS (LD): 528.2 (M^+). HRMS calculated for $C_{36}H_{36}O_2Si$: 528.2484; found: 528.2487.

Quantum Dot Synthesis

Synthesis of PbS QDs was carried out following the procedure by Hines and Scholes with modifications.[4,5] In summary, PbO (0.45 g), oleic acid (1.6 - 14 g, depending on the targeted band gap) and 1-octadecene (10 g) were degassed in a three-necked flask at 110°C for 2 h. The temperature was then reduced to 95°C. Under nitrogen, a solution of bis(trimethylsilyl)sulphide (210 µL) in 1-octadecene (5 mL) was rapidly injected into the lead precursor solution. After cooling naturally to room temperature the PbS QDs were washed 4 times by precipitation/re-dispersion with acetone and hexane. The purified QDs were stored in a nitrogen filled glovebox at high concentration (>40 mg/mL / >100 µM) until use.

Ligand Exchange

Ligand exchange was carried out under nitrogen. QDs in toluene were diluted to 42 μM in a Toluene/THF mixture of 4:1. The ligand in 100 mg/mL THF solutions was added to the QD solution. Two ligand to QD mass ratios were used for each ligand/QD pair; 1:2, and 1:8. The resulting QD/ligand absorption is shown in Figures S1-S2

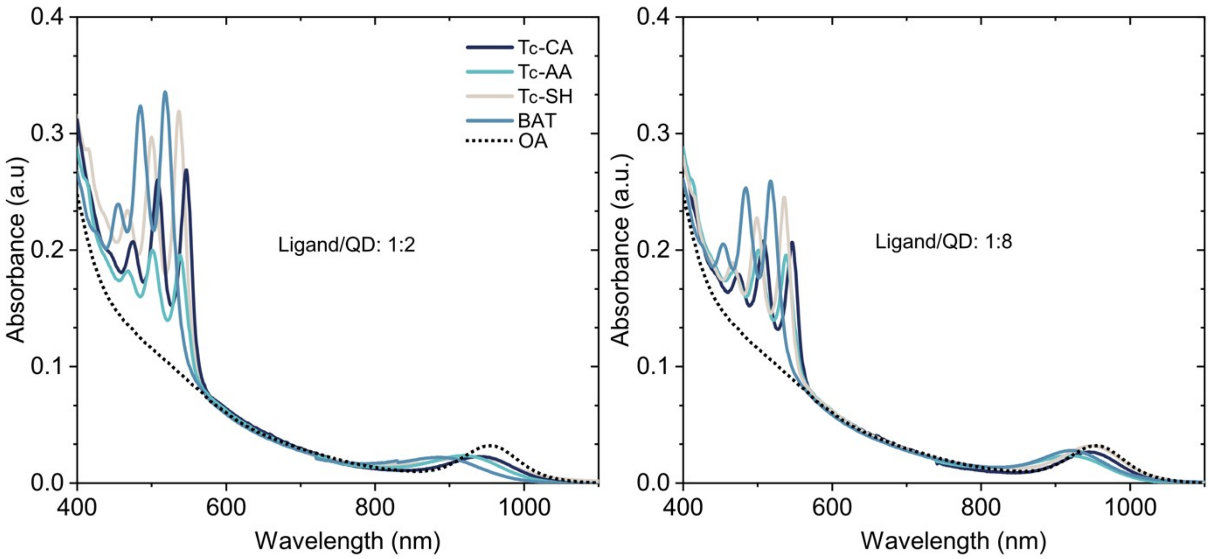

Figure S1. Absorption of PbS/ligand QD samples after ligand exchange compared to PbS/OA QDs for two different ligand loadings during ligand exchange.

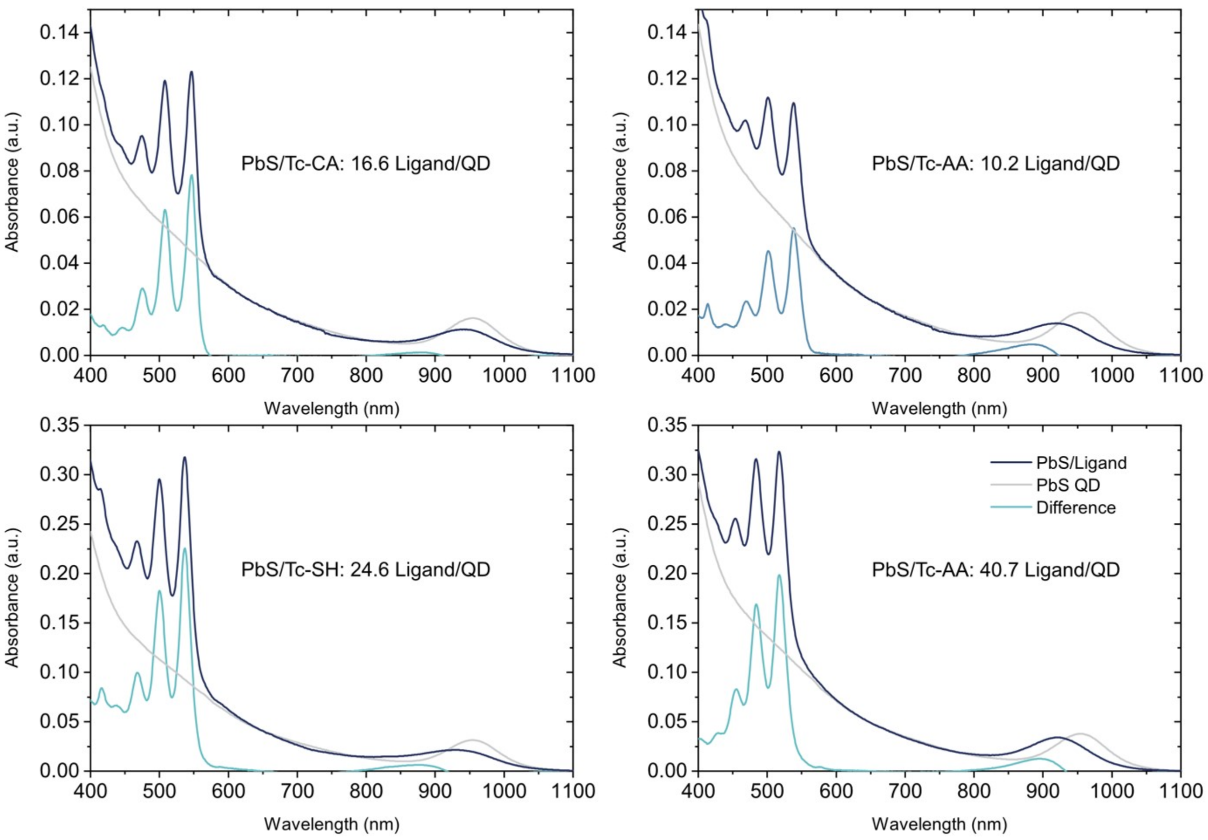

Figure S2. Absorption spectra of PbS/OA QDs and PbS/ligand QDs used to determine ligand coverage for the samples used in the transient absorption study.

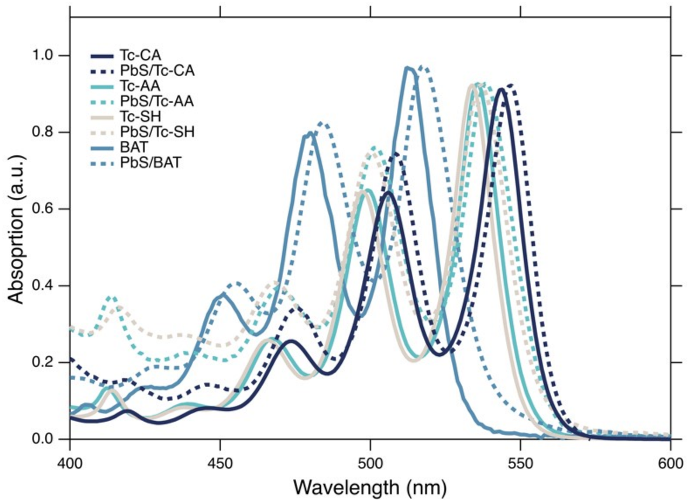

Figure S3. Comparison of the absorption spectra of the free and bound ligands.

ps-Transient Absroption

The short time (fs-ns) transient absorption setup has been described previously.[6] In summary, a Light Conversion PHAROS laser system with 400μJ per pulse at 1030 nm with a repetition rate of 38 kHz. The output is divided, one part is focused onto a 4 mm YAG substrate to produce the continuum probe beam from 520 to 950nm. The second part of the PHAROS output is lead into a narrow band optical parametric oscillator system (ORPHEUS-LYRA, Light conversion) outputting the pump beam. The probe pulse is delayed up to 2 ns with a mechanical delay-stage (Newport). A mechanical chopper (Thorlabs) is used to create an on-off pump-probe pulse series. The pump size on the sample is approximately 0.065 mm^2 and the probe about 0.015 mm^2. A silicon line scan camera (JAI SW-2000M-CL-80) fitted onto a visible spectrograph (Andor Solis, Shamrock) is used to record the transmitted probe light.

ns-Transient Absorption

The long-time ns-transient absorption setup has also been described previously.[6] In short the pump-probe setup consists of probe from a LEUKOS Disco 1 UV super continuum laser (STM-1-UV, 1 kHz) and a pump generated in a TOPAS optical amplifier, pumped with the output from a Spectra-Physics Solstice Ace Ti:Sapphire amplifier (1 kHz). The probe beam is split into a reference and probe and both are focused onto the sample. A pair of line image sensors (Hamamatsu, G11608) mounted on a spectrograph (Andor Solis, Shamrock SR303i) are used to detect the signal, using a custom-built board from Stresing Entwickslungsburo to read out the signal.

Fitting to the triplet rise and decay was done assuming that the triplet rise is described by an intrinsic transfer rate k^0_{TET}, which is the transfer to a single ligand. Assuming a Poisson distribution of bound ligands it can be shown that the transfer rate expression can be described by Equation S1[7]:

$$exp(\lambda[\exp\left(-k_{TET}^{0}t\right)-1]) \quad \text{(S1)}$$

Since the data extracted from the ns-TA experiment is an convolution of a growth term associated with triplet transfer to the ligand and the triplet decay of the ligand the fitting was done Equation S2, which incorporates both a rise term based on Equation S1 and decay term assuming a monoexponential triplet decay.

$$\frac{\Delta T}{T} = -A_1\exp\left(\lambda\left[\exp\left(-k_{TET}^{0}t\right)-1\right]\right)+A_2 exp(-k_{triplet}t) \quad \text{(S2)}$$

Cyclic Voltammetry

Cyclic voltammetry was performed on the ligands and PbS/OA QDs in an acetonitrile/toluene (1:4) mixture with $TBAPF_6$ (0.1 M) as electrolyte at a scan rate of 100 mV/s. 5 mM ferrocene was used as relative standard. A glassy carbon electrode was used as working electrode, a Pt wire as counter electrode and a $Ag/AgNO_3$ reference electrode.

Additional ps-Transient Absorption Data

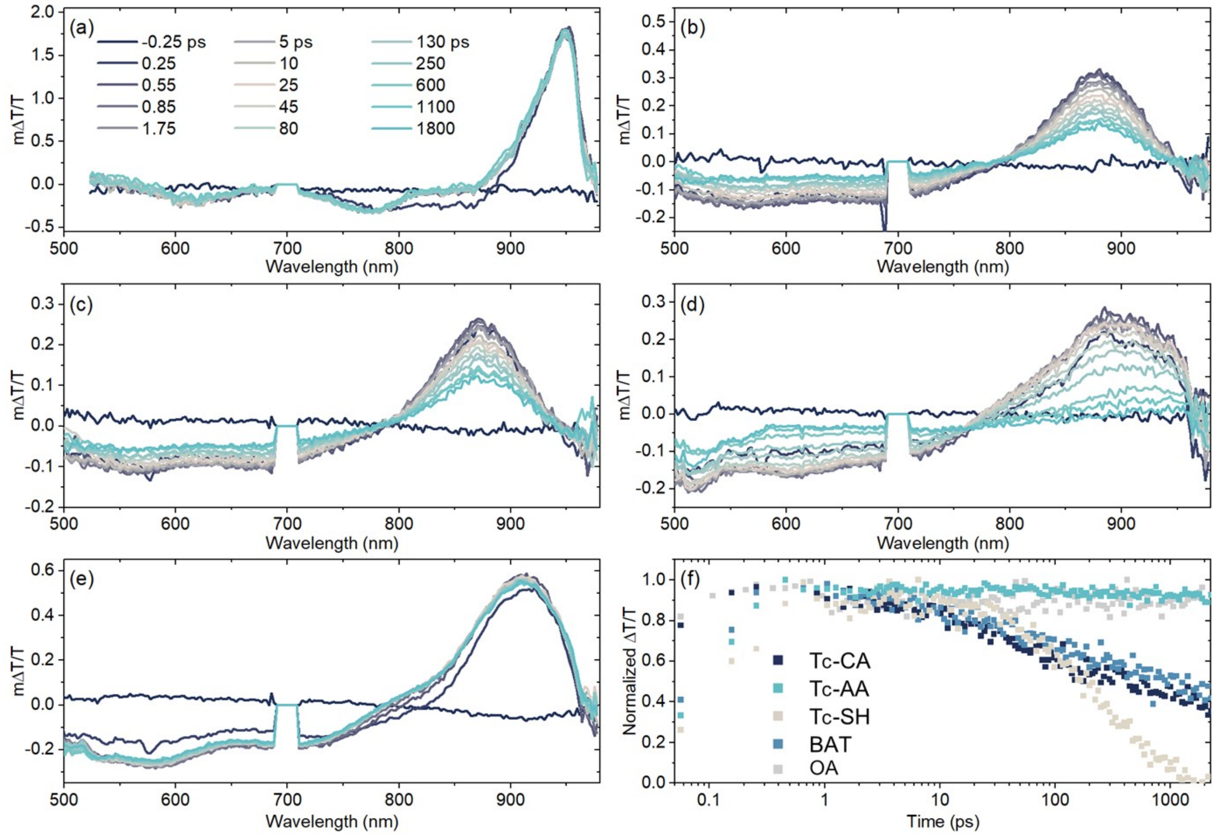

Figure S4. psTA spectra (a)-(e) for QD/ligand samples excited at 700 nm with (a) OA, (b) Tc-CA, (c) Tc-AA, (d) Tc-SH and (e) BAT ligands. (f) kinetic decay of the QD ground state bleach (GSB) in panels (a)-(e). All samples were excited at 700 nm with 10 nJ/pulse.

Additional ns-Transient Absorption Data

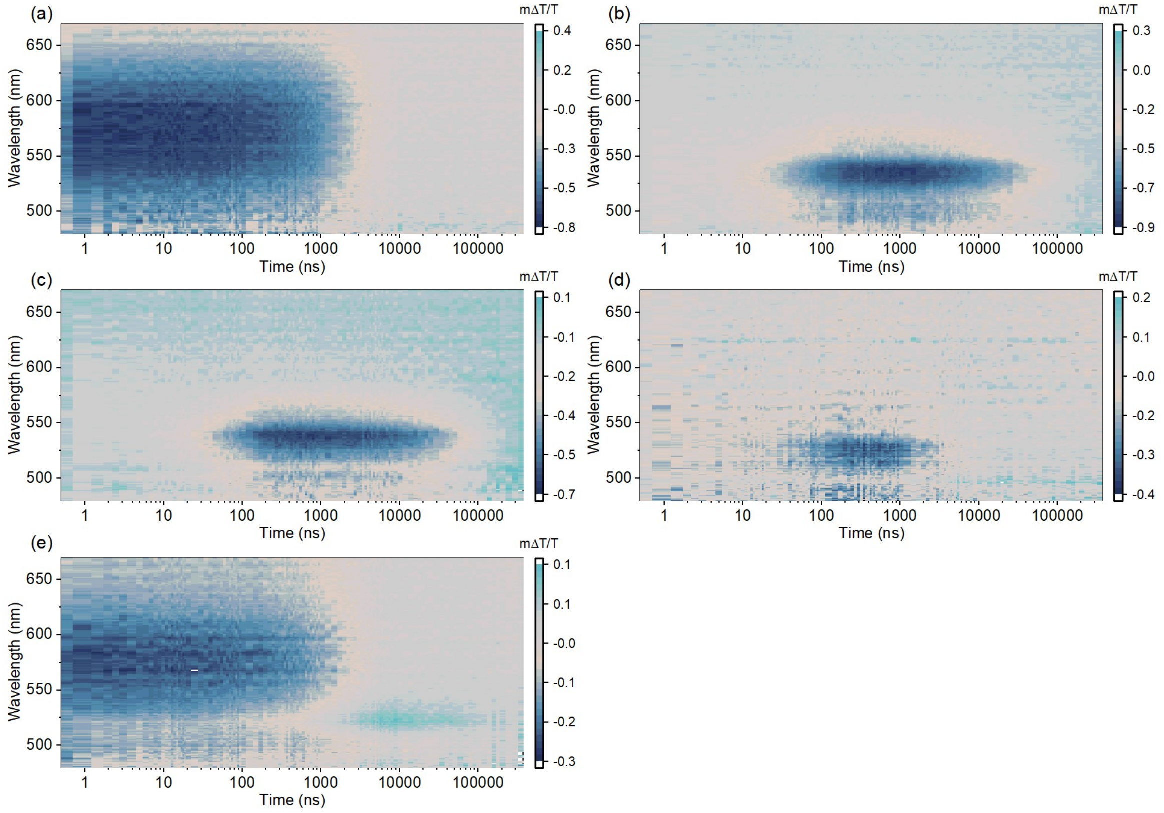

Figure S5. nsTA spectra (a)-(e) for QD/ligand samples excited at 700 nm with (a) OA, (b) Tc-CA, (c) Tc-AA, (d) Tc-SH and (e) BAT ligands. Pannels (b) and (e) are duplicates from the main text.

Cyclic Voltammograms

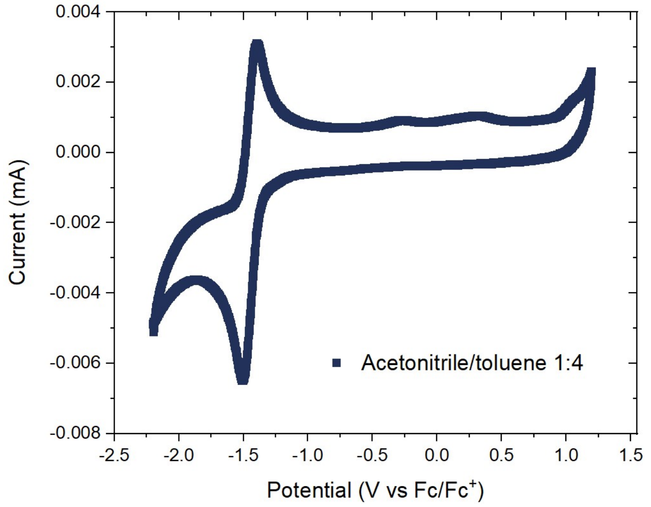

Figure S6. Cyclic voltammogram of the acetonitrile/toluene mixture with 0.1 M $TBAPF_6$ as electrolyte at a scan rate of 100 mV/s.

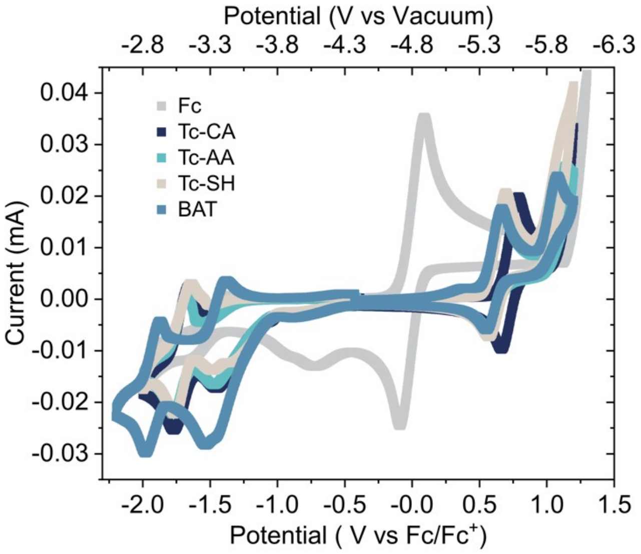

Figure S7. Cyclic voltammograms of the ligands (2mM) and ferrocene (5mM) in acetonitrile/toluene (1:4) with 0.1 M $TBAPF_6$ as electrolyte at a scan rate of 100 mV/s.

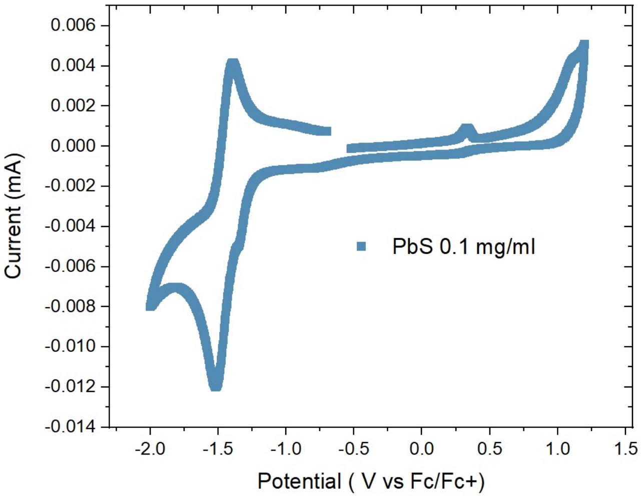

Figure S8. Cyclic voltammograms of PbS/OA (0.1 mg/ml) in acetonitrile/toluene (1:4) with 0.1 M $TBAPF_6$ as electrolyte at a scan rate of 100 mV/s.

Calculations

Structure optimization and excited state analysis

Ground state geometries are optimized at B3LYP/def2-SVP level. The dispersion correction was conducted by Grimme's D3 version by the Gaussian 16 program. Methyl groups are taken as represents in the TIPS groups of the compounds to reduce the computational costs. Basing on the optimized ground state geometries, the vertical excitation energies were evaluated at

M06-2X/def2-TZVP by TD-DFT method with polarizable continuum model (PCM) taking the dielectric constant for water. Results of these calculations are summarized in Figures S9-S12 and Table S1.

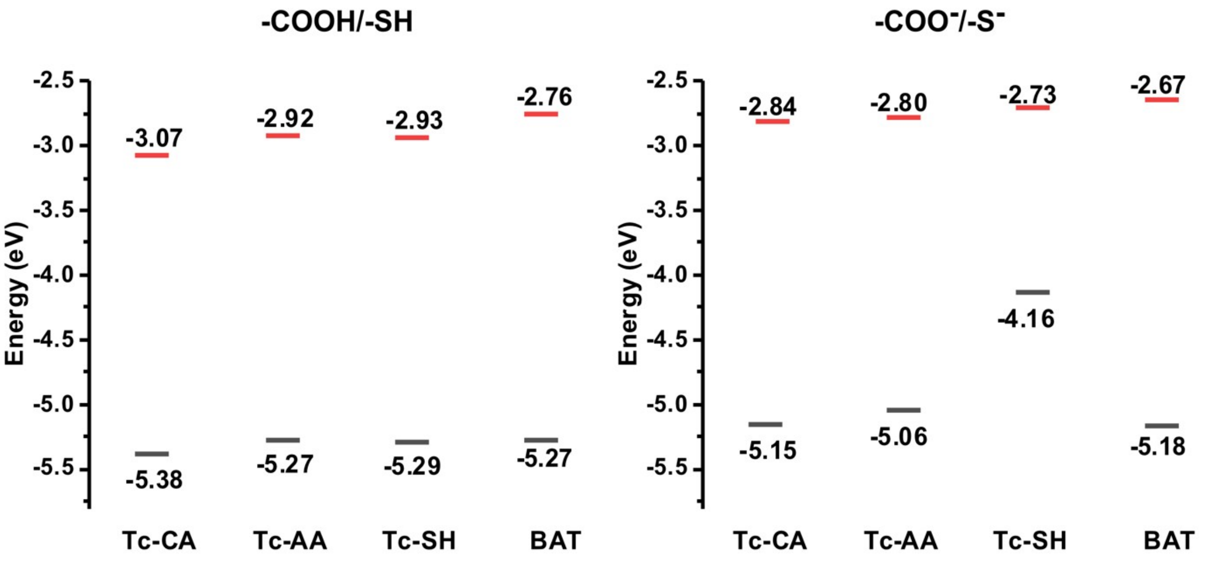

Figure S9. Frontier molecular orbitals calculated (DFT(D3)/B3LYP/def2-SVP) for the neutral (left) and deprotonated (right) forms of the ligands.

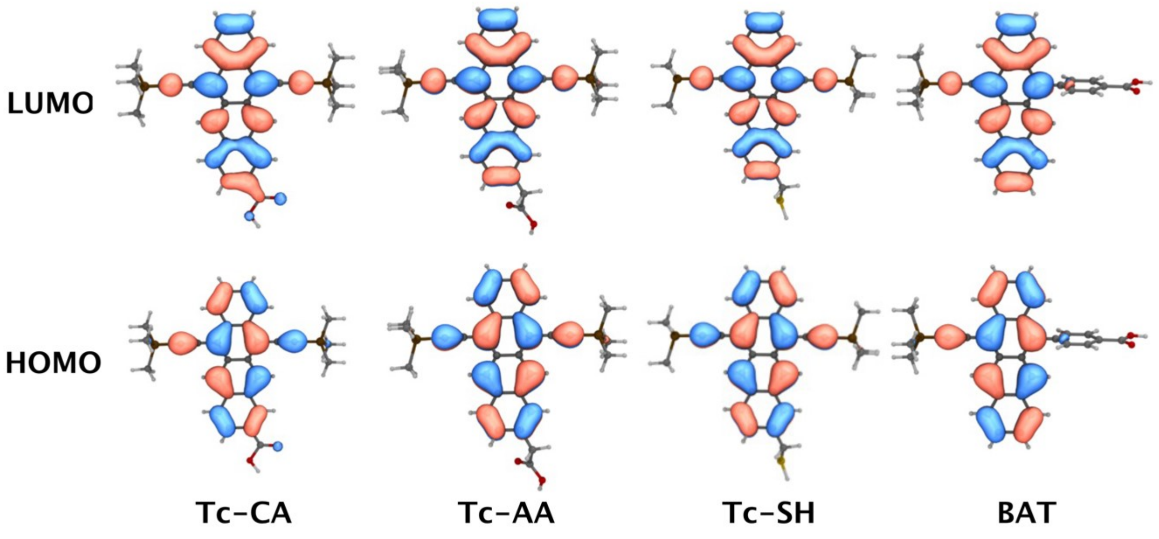

Figure S10. Distribution of the calculated frontier molecular orbitals for the neutral ligands.

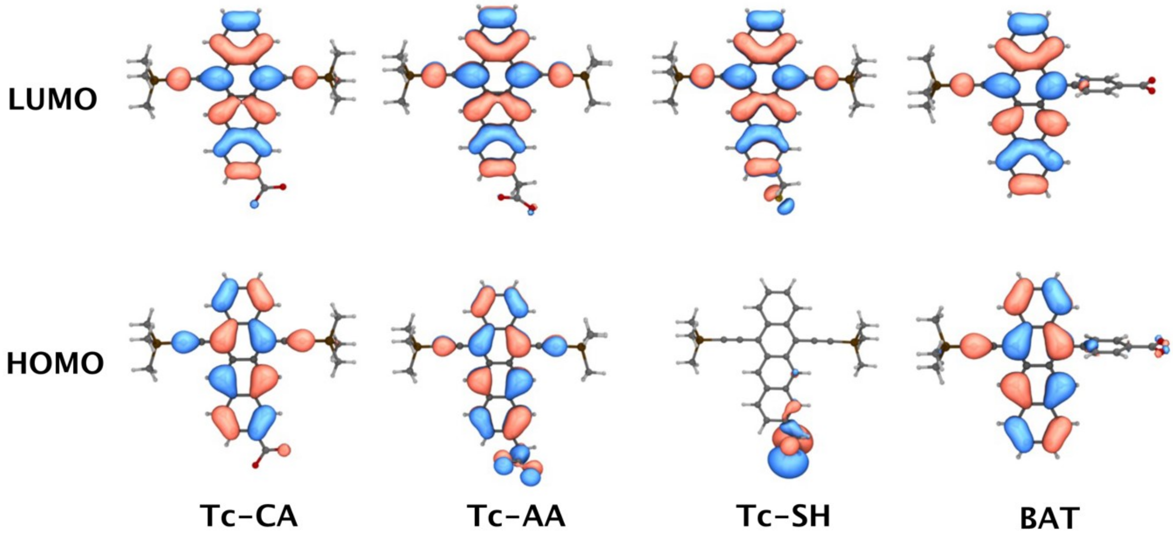

Figure S11. Distribution of the calculated frontier molecular orbitals for the deprotonated ligands.

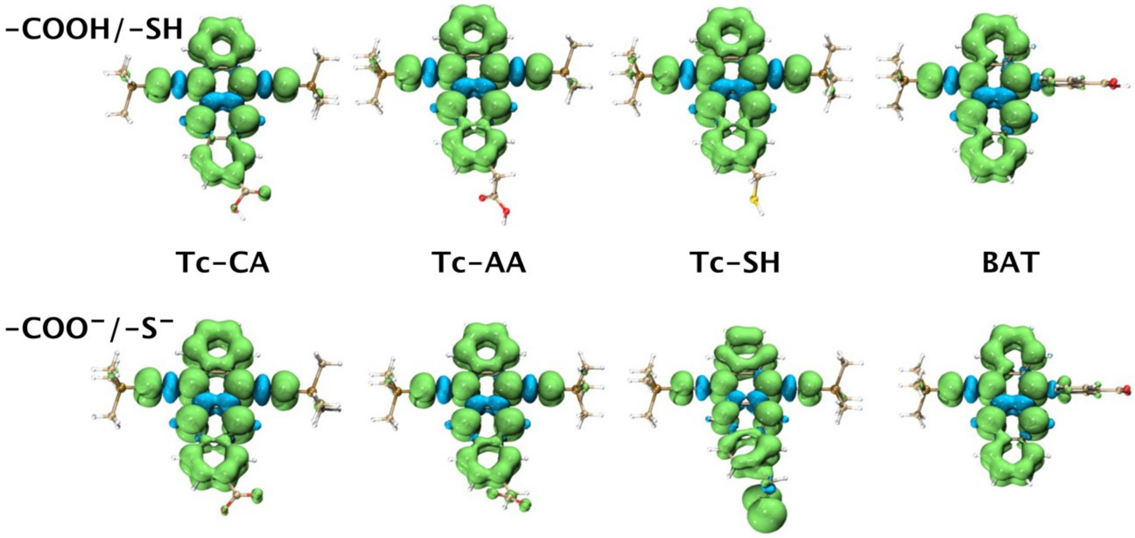

Figure S12. Electron spin density distribution of the triplet state (UB3LYP/def2-SVP) plotted for ρ_{iso} = 0.001, for the neutral (top row) and deprotonated (bottom row) forms of the ligands.

Table S1. Calculated S_1 and T_1 energies of the ligand in their neutral and deprotonated forms, compared to the experimentally determined values.

Ligand	S_1 (eV)			T_1 (eV)		
	calculated neutral	calculated deprotonated	exp. neutral	calculated neutral	calculated deprotonated	exp. neutral
Tc-CA	2.28	2.28	2.28[6]	1.09	1.11	~1.2[8]
Tc-AA	2.32	2.28	2.31[a]	1.13	1.13	

Tc-SH	2.32	2.27	2.31[a]	1.13	1.14	~1.2[2]
BAT	2.45	2.45	2.41[a]	1.26	1.26	

a) Based on the first absorption peak.

Diffusional Triplet Energy Transfer

The upconversion intensity is proportional to Equation 1 in the main text:

$$\phi_{UC} = \phi_{TET-L}\phi_{TET-A}\phi_{TTA}\phi_F \tag{S3}$$

Looking at the case where diffusional triplet transfer (TET-A) is the main reason to the upconversion difference the upconversion intensity ratio between BAT and Tc-CA samples can be rewritten as:

$$\frac{\phi_{UC-BAT}}{\phi_{UC-Tc-CA}} = \frac{\phi_{TET-A-BAT}}{\phi_{TET-A-Tc-CA}} \tag{S4}$$

To reiterate from the main text, the diffusional triplet transfer efficiency can be estimated from Equation S5:

$$\phi_{TET-A} = 1 - \frac{1}{1 + k_{TET-A}\tau_{Ligand}[Rub]} \tag{S5}$$

Using Equation S5 in Equation S4, one can solve for $k_{TET-A-Tc-CA}$, the bimolecular rate constant for triplet energy transfer from PbS/Tc-CA to Rubrene:

$$k_{TET-A-Tc-CA} = \frac{\left(\dfrac{\phi_{UC-BAT}}{\phi_{UC-Tc-CA}\left(\dfrac{\phi_{UC-BAT}}{\phi_{UC-Tc-CA}} - 1 + \dfrac{1}{1 + k_{TET-A-BAT}\tau_{BAT}[Rub]}\right)} - 1\right)}{\tau_{Tc-CA}[Rub]} \tag{S6}$$

The experimentally determined upconversion intensity ratio $\left(\frac{\phi_{UC-BAT}}{\phi_{UC-Tc-CA}}\right) = 5$, the rubrene concentration [Rub] = 10 mM in our experiments and the triplet lifetimes τ_{Ligand} are listed in Table 1 in the main text. We can then use Equation S6 to estimate the bimolecular rate constant for triplet energy transfer from PbS/Tc-CA to Rubrene to explain the experimentally observed upconversion difference for any given $k_{TET-A-BAT}$. Considering a wide range of $k_{TET-A-BAT}$ between 10^6-10^8 $M^{-1}s^{-1}$ which covers even very inefficient diffusional triplet transfer, $k_{TET-A-Tc-CA}$ has to be on the order of 10^5 $M^{-1}s^{-1}$ which is 3-4 orders below the diffusion limit in toluene. Hence, for diffusional triplet transfer to be able to explain the difference in upconversion intensity i) there has to be a 1-3 order of magnitude difference in bimolecular rate constant for triplet transfer between BAT and the three pro-*cata* ligands and ii) the pro-*cata* ligands must have a bimolecular rate constant for triplet transfer 3-4 orders below the diffusion limit. We therefore find it improbable that the diffusional triplet energy transfer from ligand to rubrene is the main reason for the observed difference in upconversion intensity. Thus, our

assumption that the upconversion intensity is directly proportional to the triplet energy transfer from QD to ligand is reasonable.

References

(1) Kroupa, D. M.; Arias, D. H.; Blackburn, J. L.; Carroll, G. M.; Granger, D. B.; Anthony, J. E.; Beard, M. C.; Johnson, J. C. Control of Energy Flow Dynamics between Tetracene Ligands and PbS Quantum Dots by Size Tuning and Ligand Coverage. *Nano Letters* **2018**, *18* (2), 865–873. https://doi.org/10.1021/acs.nanolett.7b04144.

(2) Gray, V.; Zhang, Z.; Dowland, S.; Allardice, J. R.; Alvertis, A. M.; Xiao, J.; Greenham, N. C.; Anthony, J. E.; Rao, A. Thiol-Anchored TIPS-Tetracene Ligands with Quantitative Triplet Energy Transfer to PbS Quantum Dots and Improved Thermal Stability. *The Journal of Physical Chemistry Letters* **2020**, *11*, 7239–7244. https://doi.org/10.1021/acs.jpclett.0c02031.

(3) Sanders, S. N.; Kumarasamy, E.; Pun, A. B.; Steigerwald, M. L.; Sfeir, M. Y.; Campos, L. M.; Sanders, S. N.; Kumarasamy, E.; Pun, A. B.; Steigerwald, M. L.; Sfeir, M. Y.; Campos, L. M. Intramolecular Singlet Fission in Oligoacene Heterodimers. *Angewandte Chemie - International Edition* **2016**, *128*, 3434–3438. https://doi.org/10.1002/ange.201510632.

(4) Hines, M. A.; Scholes, G. D. Colloidal PbS Nanocrystals with Size-Tunable Near-Infrared Emission: Observation of Post-Synthesis Self-Narrowing of the Particle Size Distribution. *Advanced Materials* **2003**, *15* (21), 1844–1849. https://doi.org/10.1002/adma.200305395.

(5) Zhang, J.; Crisp, R. W.; Gao, J.; Kroupa, D. M.; Beard, M. C.; Luther, J. M. Synthetic Conditions for High-Accuracy Size Control of PbS Quantum Dots. *Journal of Physical Chemistry Letters* **2015**, *6* (10), 1830–1833. https://doi.org/10.1021/acs.jpclett.5b00689.

(6) Allardice, J. R.; Thampi, A.; Dowland, S.; Xiao, J.; Gray, V.; Zhang, Z.; Budden, P.; Petty, A. J.; Davis, N. J. L. K.; Greenham, N. C.; Anthony, J. E.; Rao, A. Engineering Molecular Ligand Shells on Quantum Dots for Quantitative Harvesting of Triplet Excitons Generated by Singlet Fission. *Journal of the American Chemical Society* **2019**, *141*, 12907–12915. https://doi.org/10.1021/jacs.9b06584.

(7) Morris-Cohen, A. J.; Frederick, M. T.; Cass, L. C.; Weiss, E. A. Simultaneous Determination of the Adsorption Constant and the Photoinduced Electron Transfer Rate for a Cds Quantum Dot–Viologen Complex. *J. Am. Chem. Soc.* **2011**, *133* (26), 10146–10154. https://doi.org/10.1021/ja2010237.

(8) Gray, V.; Allardice, J. R.; Zhang, Z.; Dowland, S.; Xiao, J.; Petty, A. J.; Anthony, J. E.; Greenham, N. C.; Rao, A. Direct vs Delayed Triplet Energy Transfer from Organic Semiconductors to Quantum Dots and Implications for Luminescent Harvesting of Triplet Excitons. *ACS Nano* **2020**, *14* (4), 4224–4234. https://doi.org/10.1021/acsnano.9b09339.
